# Letter From the Editor in Chief

**DOI:** 10.19102/icrm.2018.090507

**Published:** 2018-05-15

**Authors:** Moussa Mansour


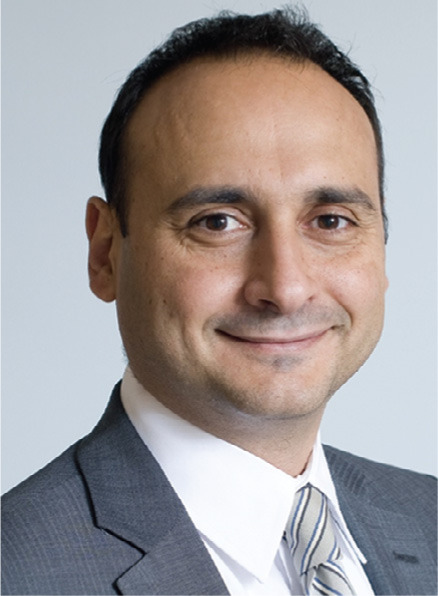


Dear Readers,

Sudden cardiac death (SCD) affects more than 300,000 people in the United States annually.^1^ The prevention of this disorder continues to be challenging despite many years of research and speculation. Numerous studies and registries have helped to identify subsets of patients with permanent electrical and anatomical pathologies who could benefit from implantable cardioverter-defibrillator (ICD) therapy. However, the optimal prevention strategies to be used in patients with a temporary risk of SCD due to reversible conditions remain elusive.

The introduction of wearable cardioverter-defibrillators represented a major step in the effort of SCD prevention. However, the data for this field are significantly less robust as compared with those for the field of implantable devices. This issue of *The Journal of Innovation in Cardiac Rhythm Management* contains an important meta-analysis by Nguyen et al.^2^ titled “Wearable Cardioverter-defibrillators for the Prevention of Sudden Cardiac Death: A Meta-analysis.” In it, the authors systematically analyzed 11 studies, most of them retrospective. They found that that the incidences of all-cause mortality and SCD-related mortality among WCD patients were very low (1.4% and 0.2%, respectively). In addition, they found that a total of 1.7% of patients received appropriate WCD treatment, and that successful ventricular tachycardia (VT)/ventricular fibrillation (VF) termination following appropriate treatment occurred in 95.5% of patients (with VT/VF having occurred in 2.6% of patients). The incidence of inappropriate treatment was infrequent (0.9%).

Since the submission and acceptance for publication of the above-mentioned meta-analysis, the results of the Vest Prevention of Early Sudden Death Trial (VEST)^3^ have also been presented. This was a multicenter, randomized trial that enrolled patients within seven days of hospital discharge after acute myocardial infarction (MI) who had an ejection fraction of 35% or less. The participants were randomized to receive either a WCD and optimal medical therapy or only optimal medical therapy. In this study, WCD did not significantly reduce sudden death mortality; however, it did reduce total mortality in the first 90 days post-MI.

In conclusion, data from one randomized study and a meta-analysis of retrospective studies appear to support the finding that the use of WCD therapy is reasonable in high-risk patients with a low left ventricular ejection fraction post-MI until evaluation for an ICD can be done at 40 days to 90 days. In addition, WCD use appears to be successful in terminating VT/VF.

I hope that you enjoy reading this issue of the *Journal*.

Sincerely,


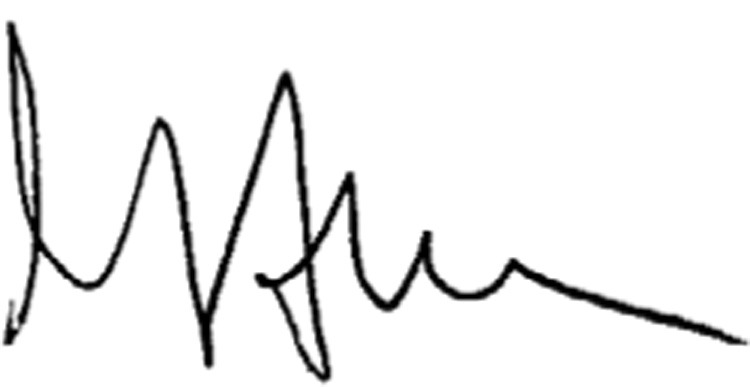


Moussa Mansour, MD, FHRS, FACC

Editor in Chief

The Journal of Innovations in Cardiac Rhythm Management

MMansour@InnovationsInCRM.com

Director, Atrial Fibrillation Program

Jeremy Ruskin and Dan Starks Endowed Chair in Cardiology

Massachusetts General Hospital

Boston, MA 02114
